# Dose–Response Relationship of Outdoor Exposure and Myopia Indicators: A Systematic Review and Meta-Analysis of Various Research Methods

**DOI:** 10.3390/ijerph16142595

**Published:** 2019-07-21

**Authors:** Ciao-Lin Ho, Wei-Fong Wu, Yiing Mei Liou

**Affiliations:** 1 School of Nursing, National Yang-Ming University, Taipei 11221, Taiwan; 2 Department of Pediatrics, Taipei City Hospital Ren-Ai Branch/Department of Allergy and Immunology, West Garden Hospital, Taipei 10864, Taiwan

**Keywords:** axial length, near-sightedness, preschool, school age, spherical equivalent refractive error (SER)

## Abstract

Myopia in children has dramatically increased worldwide. A systematic review and meta-analysis were conducted to evaluate the effects of outdoor light exposure on myopia. According to research data from 13 studies of 15,081 children aged 4–14 at baseline, outdoor light exposure significantly reduced myopia incidence/prevalence (odds ratio [OR] = 0.85, 95% confidence interval [CI]: 0.80–0.91, *p* < 0.00001; I2 = 90%), spherical equivalent refractive error (SER) by 0.15 D/year (0.09–0.27, *p* < 0.0001), and axial elongation by 0.08 mm/year (−0.14 to −0.02, *p* = 0.02). The benefits of outdoor light exposure intervention, according to pooled overall results, included decreases in three myopia indicators: 50% in myopia incidence, 32.9% in SER, and 24.9% in axial elongation for individuals in Asia. Daily outdoor light exposure of more than 120 min was the most effective intervention, and weekly intervention time exhibited a dose–response relationship with all three indicators. Subgroup comparisons revealed that interventional studies report greater benefits from outdoor light exposure compared with cohort and cross-sectional studies, and individuals with myopia in intervention studies experienced slightly greater benefits than individuals without, in terms of SER and axial elongation. Therefore, this study suggests 120 min/day of outdoor light exposure at school.

## 1. Introduction

Myopia has become a major public health problem worldwide, and its prevalence has increased rapidly, especially in East Asian countries such as Hong Kong, Singapore, South Korea, Taiwan, and China [1,2]. Myopia prevalence among children in East Asian and Western countries respectively is 80–90% [3] and 2–29.4% [1], and it is expected to increase in the future [4]. With the increase in myopia prevalence, myopia treatment and prevention have become key issues. Untreated myopia in children may lead to high myopia (over −6.0 D). High myopia is associated with ocular abnormalities such as glaucoma, retinal detachment, and myopic macular degeneration [5,6], which may seriously damage vision [7]. Related studies have reported that myopia exposes children to physical and psychological challenges as well as limitations and stigmatization for wearing spectacles [8,9], and it affects children’s learning and quality of life [10]. To avoid visual impairment, it is necessary to correct refractive errors, which represents a considerable burden. In addition to direct costs of approximately US $148 per year per child [11], other social and national costs from myopia are difficult to estimate.

The pathological changes induced by myopia in axial length and SER increase with age. Myopia management consists of a combination of drugs, glasses, contact lenses, surgery, and behavioral interventions. However, for protection against development of myopia, the most discussed behavioral intervention is outdoor light exposure. Several studies have found that outdoor light exposure is related to myopia incidence and prevalence [12,13,14,15,16], and some evidence suggests outdoor light exposure slows myopia progression. Animal experiments have demonstrated the benefits of increased ambient illumination. In chick experiments, a low-illumination environment was a risk factor for myopia [17], and high illuminance (15,000 lux) effectively reduced form deprivation myopia [18]. Another study indicated outdoor light exposure can slow form deprivation myopia in chick, macaque, and tree shrew, and it also slows lens induced myopia in chick and tree shrew under shade outdoors (about 15,000 lux on a sunny day) [19]. However, light has less effect on lens-induced myopia. By identifying the underlying mechanisms of this phenomenon in chicks, studies have found that sunlight induces dopamine release from the retina, prevents eyeball enlargement, and inhibits axial elongation. If light deprivation and low ambient illuminance inhibited the dopamine receptor in chicks, axial elongation will not be inhibited, and myopic changes will increase [20]. In addition, some studies have reported that myopia was associated with sunlight. Sunlight can promote the skin’s production of vitamin D to influence refractive development [3,6,20], stimulate retinal neurons to secrete dopamine to regulate the sclera, and prevent eye elongation [18,21,22]. Although most studies have demonstrated that physical activity is not related to myopia [13,20,21], one study indicated that outdoor time and physical activity were both confirmed to be related to myopia, and outdoor time was suggested to be more effective in preventing myopia [15].

Systematic reviews and meta-analyses have analyzed outdoor light exposure in relation to myopia prevention and control. A systematic literature review conducted by the University of Cambridge in the United Kingdom revealed that children who spend an additional hour outside each week have a 2% reduced risk of myopia. Each additional hour of daily outdoor light exposure can reduce the risk of myopia by 13% [23]. In addition, Xiong et al. [24] reviewed a total of 25 studies related to outdoor light exposure time and myopia from 2002 to 2015 in a meta-analysis and concluded that outdoor light exposure is effective only for individuals without myopia. If prevention techniques are effective only on individuals without myopia (that is, not preventing myopia progression in children with myopia), convincing policy makers to fully implement these techniques may be difficult. Therefore, the effect of outdoor light exposure on individuals with myopia requires further investigation.

Few studies have analyzed the effectiveness of outdoor light exposure intervention programs in slowing myopia progression. We therefore conducted a systematic review and meta-analysis that consider and include data from recently published cross-sectional studies, cohort studies, and intervention studies to evaluate the following six questions: Is outdoor light exposure effective in preventing myopia and in controlling myopic progression? Are intervention programs effective in reducing myopia incidence and progression? Why do distinct research methods generate dissimilar results regarding reductions in myopia incidence? Does outdoor light exposure have different effects on individuals with and without myopia? What outdoor light exposure intervention program is the most suitable for myopia prevention? Do intervention programs have a positive dose–response effect on myopia incidence and progression?

## 2. Materials and Methods

### 2.1. Inclusion Criteria

In this study, the literature collected comprised studies with participants aged 4 to 14 years at baseline, and studies investigating the relationship between outdoor light exposure time and myopia in terms of prevalence, incidence, axial length, or spherical equivalent refraction. We excluded the following: editorials, review articles, and case reports; incomplete texts; and articles not written in English or Chinese.

### 2.2. Types of Studies

Topical cross-sectional, cohort, and intervention studies with long-term follow-ups were selected. The list of eligible subject documents was searched, and duplicates or omissions were manually confirmed.

### 2.3. Search Strategy

For the purpose of this study, the systematic review of the literature was conducted using the process developed in the Cochrane Handbook for Systematic Reviews [25]. Because outdoor light exposure is a novel strategy for the prevention and treatment of myopia, it is currently a popular research topic worldwide. Therefore, a search was conducted of the Cochrane Library, MEDLINE, CINAHL, PubMed, China Academic Journals full-text database, and National Digital Library of Theses and Dissertations in Taiwan for articles published from 2000 to 2019 using the following search terms: “child”, “children”, “childhood”, “adolescent*”, “teen*”, “pediatric”, “paediatric”, “youth*”, “outdoor*”, “myopia*”, “refractive*”, and “nearsightedness*”. Each primary article obtained from the search was studied to determine its potential for inclusion. Article selection was conducted according to the PRISMA (Preferred Reporting Items for Systematic Reviews and Meta-analysis) guidelines.

### 2.4. Assessment of Methodological Quality

To comply with methodological quality, the literature was evaluated, scored, analyzed, and extracted according to participant inclusion and research questions designed by two independent reviewers. After evaluation for comparison and integration, the results were ultimately confirmed by the corresponding author. The quality of the intervention studies was assessed using the Cochrane bias risk assessment tool, which comprises seven aspects as follows: (1) random sequence generation (selection bias), (2) allocation concealment (selection bias), (3) blinding of participants and researchers (performance bias), (4) blinding of outcome assessment (detection bias), (5) incomplete outcome data (attrition bias), (6) selective reporting (reporting bias), and (7) other errors. According to the scoring standard, “+” represents a low risk of bias, “?” refers to an unclear risk of bias, and “−” indicates a high risk of bias [25]. The quality of the cohort studies and cross-sectional studies was assessed based on the standard Newcastle-Ottawa Scale (NOS). The NOS used a ‘star system’ which was applied to judge three broad perspectives: The selection of the study groups, the comparability of the groups, and the exposure/outcome. The assessment of the cohort studies had maximum 4 stars for selection, maximum 2 stars for comparability, and maximum 3 stars for exposure/outcome. The assessment of the cross-sectional studies had maximum 5 stars for selection, maximum 2 stars for comparability, and maximum 3 stars for exposure/outcome. The NOS identifies ‘high’ quality choices with a ‘star’ [26,27].

### 2.5. Data Analysis

The main objective of data extraction and meta-analysis was to assess the results of the literature. We used odds ratios (ORs) with 95% confidence intervals (CIs) and the mean difference in SER and axial length to assess myopia incidence and prevalence as well as myopia progression. For cross-sectional studies, the outdoor light exposure time was measured in hours per day, which was standardized to hours per week by multiplying the log of the OR and standard error (SE) by 7 [23]. We estimated the SE of log OR by dividing the width of the CI by 2 × 1.96 [28].

In cohort studies, we compared groups with high and low outdoor light exposure times. Intervention studies were defined as having 1-year follow-up periods, and all their follow-ups were standardized into 1-y periods. The subgroup analysis considered a myopia group, a nonmyopia group, and the overall group. The SER and axial elongation were estimated at 3-year follow-up periods [29], and the data from the studies within a 1-year follow-up were divided by a factor of 2.3 [30]. The RevMan statistical software program version 5.3.5 (Biostat, Englewood, NJ, USA), provided by the Cochrane Collaboration, was used for the meta-analysis. The χ^2^ test was used to determine the heterogeneity among the results of the included studies. When statistical homogeneity was detected among the studies (I^2^ > 50%), the meta-analysis was performed using a random-effect model. For dose–response analysis, a simple linear regression model was used to explore the relationship between outdoor light exposure time at school and myopia indicators.

## 3. Results

### 3.1. Search Results and Article Selection

**Figure 1:**
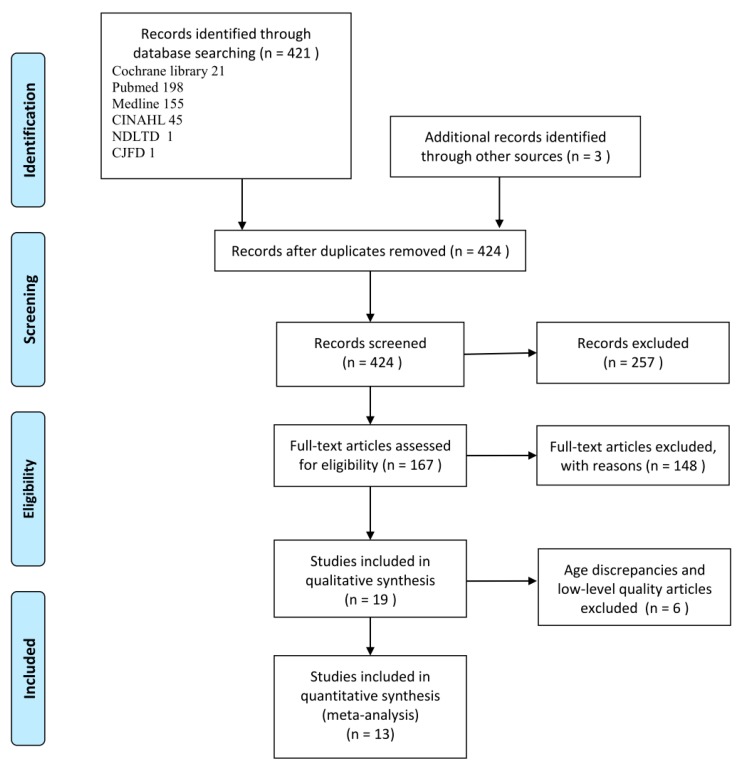
Flow chart of the literature search and study selection.

We identified 421 articles from the electronic databases and three articles from other sources. The main reasons for excluding a study were duplication or inconsistency between the title and the abstract, no full text or unavailable full text, ages outside our range, no data matching the research purposes, and poor quality of literature. After exclusion based on these criteria, 13 studies remained for analysis: Four cross-sectional studies [31,32,33,34], three cohort studies [12,15,35], and six intervention studies [29,36,37,38,39,40]. Figure 1 provides a flow chart of the literature verification and the process of screening for inclusion in the research.

### 3.2. Description of the Included Articles

The relevant features of the 13 articles are presented in Table 1; a total of 15,081 children were enrolled. Briefly, these articles comprised four cross-sectional studies (5745 participants), three cohort studies (4622 participants), and six intervention studies (4714 participants). Participants in these studies were school-aged children between 4 and 14 years old at baseline. The selected studies were from China, Taiwan, Australia, the United Kingdom, and the United States. The articles included were published between 2000 and 2019, in accordance with our criteria. All databases were searched, and the validity of the qualified studies was critically appraised. On the basis of the inclusion criteria, 13 articles were judged to be of moderate to high quality (Tables S1 and S2, Figures S1 and S2 in Supplementary Materials). A total of 12 articles used questionnaires to collect the weekly [12,16,31,34,35,36,37,40] or daily outdoor time [15,33,38,39]. Only one article used light meters to objectively measure outdoor time [39]. The intervention types and increased outdoor times at school are presented in Table 2.

### 3.3. Association Between Outdoor Time and Risk of Incident/Prevalent Myopia

**Figure 2:**
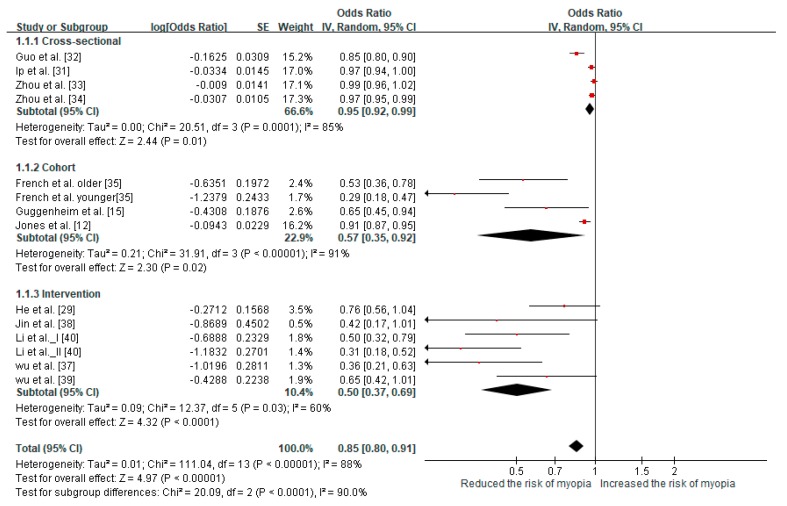
Results of the meta-analysis for myopia incidence and prevalence.

The pooled meta-analysis investigated associations of the incidence and prevalence of myopia with outdoor time, as illustrated in Figure 2. The data of twelve articles corresponded to incident/prevalent myopia and were categorized into three subgroups: Cross-sectional studies, cohort studies, and intervention studies. One of the cohort studies used a younger group and an older group [35], and one of the intervention studies used a control group and two groups receiving distinct interventions [40]. An I^2^ value ≥50% indicates high statistical heterogeneity; therefore, the random-effect model was used for the study of the three subgroups. Some data measured in h/day were converted into standardized effect estimates in h/week.

The cross-sectional studies were pooled and yielded an OR of 0.95 (95% CI: 0.92–0.99) for myopia prevalence per additional hour of exposure per week. Comparing the high-level and low-level exposure time groups from the cohort studies revealed that the outdoor time of the high-level group resulted in a significantly reduced risk of myopia incidence (OR = 0.57, 95% CI: 0.35–0.92). The intervention studies revealed that outdoor light exposure time had a significant protective effect on the risk of myopia incidence (OR = 0.5, 95% CI: 0.37–0.69). We applied a random-effect model to analyze the three subgroups because the I^2^ value was ≥50%. The overall meta-analysis yielded a pooled OR for myopia of 0.85 (95% CI: 0.80–0.91) per additional hour of time spent outdoors per week.

### 3.4. Reducing SER

**Figure 3:**
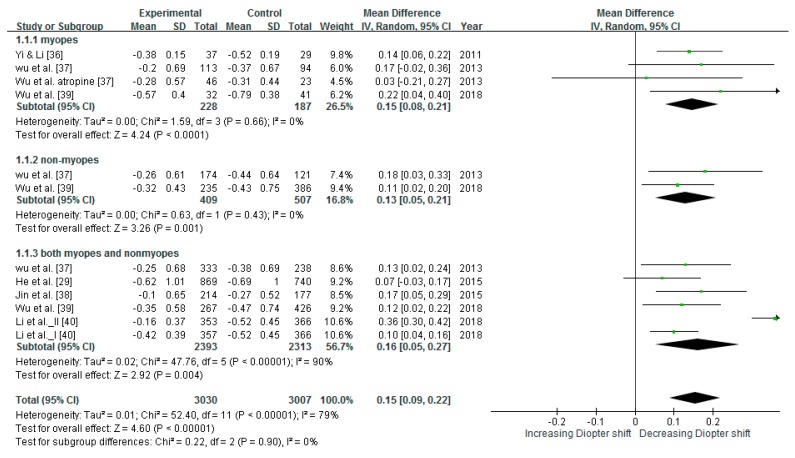
Results of meta-analysis for SER after interventions.

Six intervention studies of a total of 4,406 children were included in meta-analysis of effects of SER. Most of the included intervention studies had a control group and an intervention group. However, one study had a control group and two intervention groups [40]. Group I was assigned 7 h/week of exposure, and Group II was assigned 1-h outdoor light exposure after school; the control group received no outdoor light exposure intervention. The heterogeneity test result of the studies was I^2^ = 79% (*p* < 0.00001), indicating heterogeneity significance. Therefore, this study used a random-effect model to interpret the conclusion of the meta-analysis. The pooled estimates indicated that the mean difference in the change of SER showed significantly reduction (mean difference = 0.15 D, 95% CI: 0.09–0.22) in Figure 3. The mean differences in the change of SER for children without and with myopia were 0.13 and 0.15 D/year, respectively, and 0.16 D/year for both.

### 3.5. Slowing Axial Elongation

**Figure 4:**
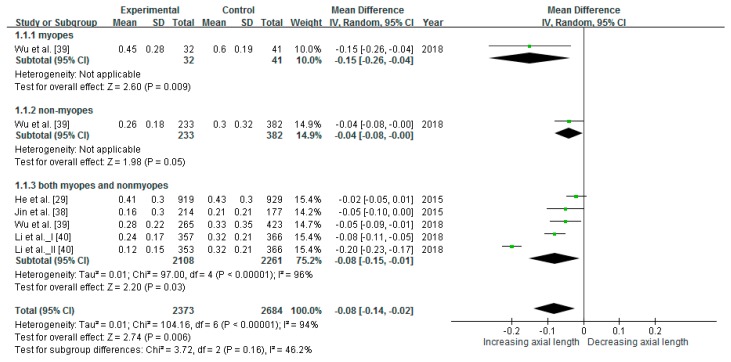
Results of the meta-analysis for axial elongation after intervention.

The intervention studies in this meta-analysis for axial elongation included a total of 3903 children. The heterogeneity test result I^2^ = 94% (*p* < 0.00001) indicated significant and high heterogeneity among the studies. Therefore, a random-effect model was used to interpret the conclusion of the meta-analysis, and the results revealed that outdoor light exposure slowed axial elongation. The pooled estimates indicated that the mean difference in axial elongation was statistically significant (mean difference = −0.08 mm, 95% CI: −0.14 to −0.02), as illustrated in Figure 4. The mean difference in the change of axial elongation was reduced in children without and with myopia by 0.04 and 0.15 mm/year, respectively, and 0.08 mm/year for both.

### 3.6. Effect of Reducing Myopic Progression in Intervention Studies

To standardize the effect, this study defined the ratio of myopia incidence reduction and myopia progression in terms of SER and axial length by calculating the difference between the intervention group and the control group and dividing it by the value of the control group. The ratios for the intervention studies are summarized in Table 2. Compared with control groups, the percentages of reduction were 50% (incidence/prevalence of myopia), 32.9% (SER), and 24.9% (axial length) in the intervention groups.

### 3.7. Dose–Response Effect of Intervention Programs

To analyze the effect of the interventions for outdoor light exposure time on the prevention of myopia, this study calculated the outdoor light exposure time at school. The outdoor light exposure time per school week (5 days) at school comprised class recess time, physical education time, and additional outdoor time (see Table 2). Three studies did not specify the outdoor light exposure time at school; therefore, they were not included in this section for the dose–response analysis [29,36,38]. Group I had 7 h/week of exposure—including recess and physical education—and Group II had an extra 5 h/week after school [40].

**Figure 5:**
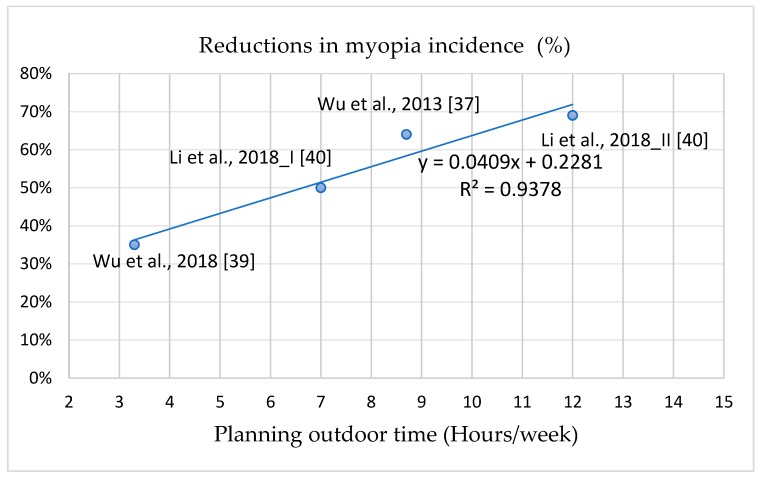
The dose-response effect between reduction ratios in incident/prevalent myopia and outdoor time at school.

**Figure 6:**
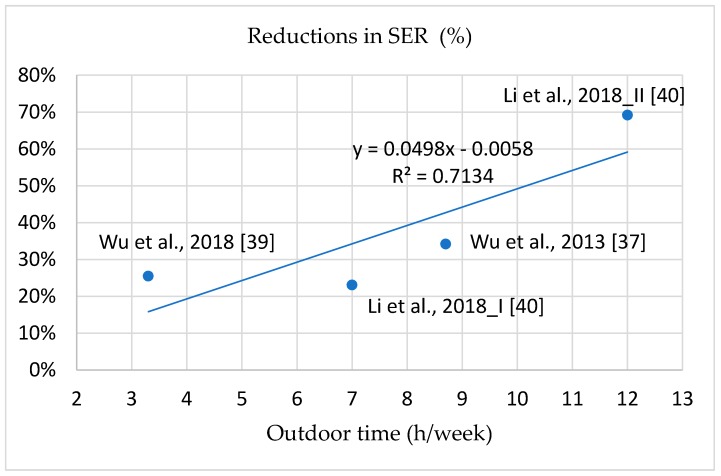
The dose-response effect between reduction ratios in SER and outdoor time at school.

**Figure 7:**
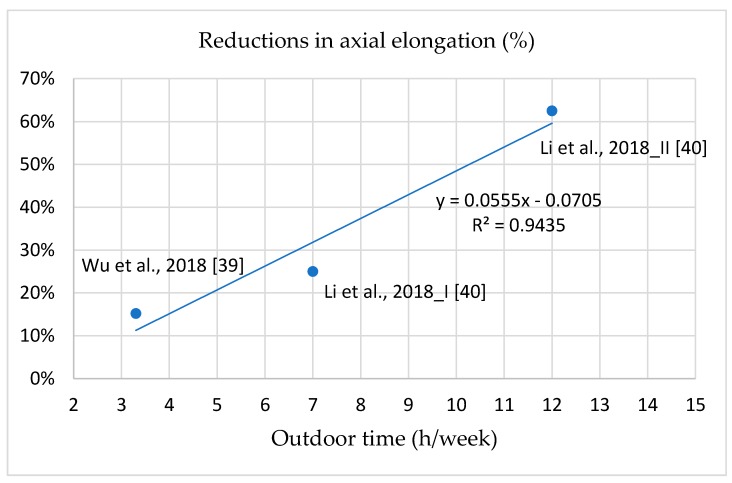
The dose-response effect between reduction ratios in axial elongation and outdoor time at school.

The correlations between outdoor light exposure time at school and decreases in myopia incidence, SER, and axial elongation are illustrated in Figure 5, Figure 6 and Figure 7; the curves are all linear. The interpretations of the variance of the benefits in the reductions of myopia incidence, SER, and axis elongation predicted by the outdoor light exposure time were 93.78%, 71.34%, and 94.35%, respectively. These results indicate that longer outdoor light exposure time at school was related to a stronger prevention effect against myopia incidence, SER, and axial elongation. Compared with the control groups, 50% lower myopia incidence, SER, and axis elongation required increases of 7, 10, and 10 h/week, respectively, of outdoor light exposure time at school.

## 4. Discussion

### 4.1. Outdoor Light Exposure Prevented Myopia and Slowed Myopic Progression

This study used a systematic review and meta-analyses to evaluate the relationship between outdoor light exposure and myopia from 13 articles concerning children 4 to 14 years old at baseline, including cross-sectional, cohort, and intervention studies. However, the participants in the six intervention studies were all Chinese, whereas the participants in the other studies included East Asian, Caucasian, and Hispanic ethnic groups. The results of this study indicate that the outdoor light exposure programs of the intervention studies, compared with control groups, are the most effective for the prevention and control of myopia and may significantly reduce myopia incidence and SER and axial length.

### 4.2. Cross-Methodology Validated the Reduction in Myopia Incidence/Prevalence

To rigorously analyze the effects of outdoor light exposure on myopia, this study used cross-methodology on three research categories, namely cross-sectional studies, cohort studies, and intervention studies. That is, three subgroup analyses of the incidence and prevalence of myopia were performed in this study. The results indicated that the OR of myopia incidence/prevalence was 0.95 in the cross-sectional studies, 0.57 in the cohort studies, and 0.5 in the intervention studies. Outdoor light exposure time appeared to affect the prevalence of myopia slightly in cross-sectional studies. Among the cohort studies, the OR reported by French et al. of the younger cohort (age 6 years at baseline) was the smallest (0.29); therefore, long outdoor light exposure (>23 h) may be more efficient in reducing myopia incidence for younger children [35]. The benefit of longer outdoor light exposure time induces a significant reduction in myopia incidence and prevalence in children. Moreover, this study’s result is consistent with that of previous studies [23,41]. Compared with the cross-sectional studies and cohort studies, intervention studies exhibited stronger effects because the behavioral changes in outdoor light exposure persisted through follow-ups of 1–3 year, resulting in the intervention groups having likely received more outdoor light exposure time than the control groups. In summary, long-term outdoor light exposure should be used with interventional procedures (at school) to slow myopia progression.

### 4.3. Intervention Programs are Effective in Reducing Myopia Incidence and Progression

To compare intervention programs, this study standardized the effects on myopia incidence and myopia progression in terms of SER and axial length. The estimated overall reduction in myopia incidence was 50%, and this result is similar to the results of other meta-analyses [23,24]. In contrast to previous studies, this study included six studies for additionally analyzing myopic shifts in SER and axial length. Reductions compared with the control groups in SER and axial elongation were 32.9% and 24.9%, respectively.

Axial length is highly correlated with SER and is associated with myopia progression [42,43]. Therefore, outdoor light exposure programs can not only significantly reduce myopia incidence but also slow myopic shift. According to the aforementioned results, outdoor light exposure intervention is a positive strategy for myopia prevention in children, and it warrants promotion.

### 4.4. Outdoor Light Exposure Intervention Reduces Myopia Progression in Children with Myopia and Prevents Myopia Development in Children Without Myopia

The intervention groups in this study exhibited significantly less myopic shift compared with the control groups. In the intervention groups, the reductions in the mean difference in the change of SER for children without and with myopia were 0.13 and 0.15 D/year, respectively, and 0.16 D/year overall. In the intervention groups, the mean difference in the change of axial elongation was reduced for children without and with myopia by 0.04 and 0.15 mm/year, respectively, and 0.08 mm/year overall. The myopic shift in children with myopia decreased more than that of children without myopia. Although a previous study indicated that outdoor time in ineffective in slowing myopic progression in individuals with myopia [24], the overall analysis of this study revealed a significant reduction thereof in individuals with myopia. The number of research articles or of individual myopia cases in the previous study may have been insufficient. In this study, the analysis of changes in axial length assessed only one study including individuals both with and without myopia [29], and this analysis did not yield significant results; however, the overall analysis indicated a significant difference in axial elongation. Therefore, outdoor light exposure intervention programs appear to have stronger preventive and therapeutic effects on individuals with myopia than on individuals without. Individuals both with and without myopia exhibited significantly less myopic shift, indicating that outdoor light exposure is effective regardless of myopia status; therefore, such interventions should be promoted for all children.

### 4.5. Positive Dose–Response Effect of Intervention Programs on Myopia Incidence and Progression

The most effective intervention program in reducing myopia incidence, SER, and axial elongation was intervention Group II in the study of Li et al., in which the ratios were reduced by 69%, 69.2%, and 62.5% compared with the control group [40]. The intervention Group II had 7 h/week of exposure and an extra 5 h/week after school. The threshold duration of outdoor light exposure required to prevent myopia is unknown, despite numerous studies attempting to determine it. Studies have indicated that individuals without myopia experience 11.7–21 h/week of outdoor light exposure [12,34,35,41]. This wide time range and inconsistency may have been caused by inaccurately recorded durations or other factors. However, because school intervention programs can be easily controlled by school administration, the actual outdoor time during school is controllable. This study demonstrated a positive dose–response effect of outdoor light exposure time at school on myopia incidence, SER, and axial length [37,39,40]. Represented by linear curves, 50% reductions in myopia incidence, myopic refraction, and axial elongation required an increase of 7, 10, and 10 h/week of outdoor light exposure time compared with control groups. We suggest 10 h/week of outdoor light exposure time at school for myopia prevention; 10 h/week, or 120 min/day, can reduce myopia incidence by 63.7%. According to the aforementioned results, we suggest that the appropriate program include compulsory outdoor recess (5 h/week), physical activity class (2 h/week), and 3 h/week of additional outdoor light exposure at school. In addition to increasing outdoor light exposure time, school intervention programs can encourage students to develop habits that increase outdoor light exposure.

### 4.6. Limitations

Although we conducted an extensive search of the databases, we found only six intervention studies of outdoor light exposure, among which only four measured axial length, two used objective tools to measure outdoor light exposure, and one contained exercise strategies. Moreover, the collected studies included participants from East Asian, Caucasian, and Hispanic ethnic groups, whereas the six intervention studies were conducted only in Asia. To establish more generalizable scientific evidence, future research should refer to the results of this study to develop and conduct a large-scale, multinational, blinded, and randomized controlled trial including Asian children and children from other ethnic groups. Another limitation of the current study would be the subjectivity of the measures from all the cross-sectional and cohort studies, because children’s weekly [12,16,31,34,35] or daily outdoor time [15,33] values in the questionnaires were provided by parents. This may also lead to a risk of recall bias and overestimate. Future studies should also add an objective measurement of the exercise strategy to facilitate comparisons and increase the extrapolation of research results.

## 5. Conclusions

This study was the first to analyze the dose–response effect relationship between outdoor light exposure time and axial length through meta-analysis. This study used a systematic review and meta-analysis to evaluate reports of outdoor time, incidence/prevalence of myopia, SER, and axial elongation in children aged 4 to 14 years at baseline. Outdoor light exposure was concluded to slow myopic progression in individuals with myopia. Outdoor intervention programs can significantly reduce myopia incidence by approximately 50% as well as slow myopic SER progression by 32.9% and axial elongation by 24.9% for individuals in Asia. The most effective outdoor intervention program combines outdoor light exposure during recess time and one additional hour of outdoor light exposure after school. Therefore, we recommend the widespread promotion of outdoor light exposure in schools in Asia, and we suggest that future studies include children from other ethnic groups.

## Figures and Tables

**Table 1:** Characteristics of the studies included in the meta-analysis of outdoor light exposure and myopia.

Study	Participants	Outcome: Myopia Incidence, Myopia Prevalence, SER, and Axial Length
**Cross-sectional studies**		
Zhou et al., 2014 [33]	823 children aged 6–12; Lanzhou City, Gansu Province, China.	Prevalence: OR = 0.937 (0.775–1.134) (h/day) transforms into OR = 0.991 (0.964–1.018) (h/week).
Zhou et al., 2015 [34]	1902 urban primary school children; mean age: 9.8 years; Guangzhou, China.	Prevalence: OR = 0.97 (0.95–0.99), h/week.
Guo et al., 2013 [32]	681 primary school students aged 8–13 years, mean age: 9.4 years; Beijing, China.	Prevalence: OR = 0.32 (0.21–0.48) (h/day) transforms into OR = 0.85 (0.8–0.902) (h/week).
Ip et al., 2008 [31]	2339 school children; mean age: 12 y; Sydney, Australia.	Prevalence: OR = 0.97 (0.94–0.995), h/week.
**Cohort studies**		
French et al. 2013 [35]	2103 students; 6 and 12 years at baseline; 5–6-year follow-up period; Australia.	Incident myopia of younger cohort (6 years old): High 8.2% (n = 22) versus low 23.3% (n = 64), OR = 0.29 (0.18–0.5), h/week. Incident myopia of older cohort (12 years old): High 15.5% (n = 52) versus low 25.8% (n = 77), OR = 0.53 (0.36–0.78), h/week. Outdoor time per week is as follows: Younger cohort, low-level (<16 h/week) and high-level (>23 h/week); older cohort, low-level (<13.5 h/week) and high-level (>22.5 h/week).
Guggenheim et al., 2012 [15]	2005 children aged ≥7 years who attended follow-up for an average of 4 years; United Kingdom.	Prediction of incident myopia: Time outdoors (high versus low), OR = 0.65 (0.45–0.96), h/week. Amount of outdoor time per week was considered high-level if the response was “3 or more h/day”; otherwise it was considered low-level.
Jones et al., 2007 [12]	514 children aged 8 or 9 years; 5-year follow-up period; United States.	The nonmyopia group spent on average 11.65 ± 6.97 h/week (high-level) in sports and outdoor light exposure, whereas the future myopia group spent an average of 7.98 ± 6.54 h/week (low-level) outdoors. Outdoor time (nonmyopia versus myopia), OR = 0.91 (0.87–0.94), h/week.
**Intervention studies**		
Yi & Li, 2011 [36]	80 children with myopia aged 7–11 years; 2-year follow-up period; China.	An intervention group (n = 41) and a control group (n = 39). Myopia group: Intervention: −0.38 ± 0.15 D/year, n = 37; control: −0.52 ± 0.19 D/year, n = 29.
Wu et al., 2013 [37]	571 children aged 7–11 years; 1-year follow-up period; Kaohsiung, Taiwan.	New cases of myopia onset, intervention group vs. control group: 8.41% (28/174) vs. 17.65% (42/121); *p* < 0.001. Nonmyopia group, intervention: −0.26 ± 0.61 D/year, n = 174; control, −0.44 ± 0.64 D/year, n = 121. Myopia group without atropine treatment: Intervention, −0.20 ± 0.69 D/year, n = 113; control, −0.37 ± 0.67 D/year, n = 94. Myopia group with atropine treatment: Intervention, −0.28 ± 0.57 D/year, n = 46; control, −0.31 ± 0.44 D/year, n = 23. Both myopia and nonmyopia group: Intervention, −0.25 ± 0.68 D/year, n = 333; control, −0.38 ± 0.69 D/year, n = 238.
Jin et al.,2015 [38]	391 children; grades 1, 3, 5, and 7; urban and rural; 1-year follow-up; Northeast China.	Incidence of new myopia onset, the intervention group vs. the control group: 3.70% (8/214) vs. 8.50% (15/177), *p* = 0.048. Both myopia and nonmyopia group: Intervention, −0.10 ± 0.65 D/year, 0.16 ± 0.30 mm/year, n = 214; control, −0.27 ± 0.52 D/year, 0.21 ± 0.21 mm/year, n = 177.
He et al., 2015 [29]	1903 children; mean age: 6.6 years; 3-year follow-up; Guangzhou, China.	Cumulative incidence rate, intervention group vs. control group: 30.4% (259/853) vs. 39.5% (287/726)/3 years transforms into 10.1% (86/853) vs. 13.2% (96/ 726)/year. Both myopia and nonmyopia group: Intervention: −1.42 (−1.58 to −1.27)/3 years transforms into −0.62 ± 1.01 D/year, n = 869; 0.95 (0.91 to 1.00) mm/3 years transforms into 0.41 ± 0.30 mm/year, n = 919. Control: −1.59 (−1.76 to −1.43)/3 years transforms into −0.69 ± 1.00 D, n = 740; 0.98 mm (0.94 to 1.03)/3 years transforms into 0.43 ± 0.30 mm/year, n = 929.
Wu et al., 2018 [39]	693 children aged 6–7 years; 1-year follow-up; northern, central, southern, and western Taiwan.	Incidence of new myopia onset in the intervention group was less than that in the control group (14.47% vs. 17.40%), and risk of myopia was 35% lower (OR = 0.65; 95% CI: 0.42–1.01; *p* = 0.054). Nonmyopia group: Intervention: −0.32 ± 0.43 D/year, 0.26 ± 0.18 mm/year, n = 235; control: −0.43 ± 0.75 D/year, 0.3 ± 0.32 mm/year, n = 386. Myopia group: Intervention: −0.57 ± 0.4 D/year, 0.45 ± 0.28 mm/year, n = 32; control: −0.79 ± 0.38 D/year, 0.6 ± 0.19 mm/year, n = 41. Both myopia and nonmyopia group: Intervention: −0.35 ± 0.58 D/year, n = 267; 0.28 ± 0.22 mm/year, n = 265; control: −0.47 ± 0.74 D/year, n = 426; 0.33 ± 0.35 mm/year, n = 423.
Li et al., 2018 [40]	1076 children aged 6–8 years; 1-year follow-up; Wenzhou area, China. 366 participants in the control group, 357 participants in test Group I, and 353 participants in test Group II.	Cases of newly onset myopia, intervention groups vs. control group: 32 (32/357) (Group I), 20 (20/353) (Group II), 60 (60/366). Refractive error changes, axial changes Both myopia and nonmyopia, Group I: Intervention: −0.42 ± 0.39 D/year, 0.24 ± 0.17 mm/year, n = 357; control: −0.52 ± 0.45 D/year, 0.32 ± 0.21 mm/year, n = 366. Both myopia and nonmyopia group, Group II: Intervention: −0.16 ± 0.37 D/year, 0.12 ± 0.15 mm/year, n = 353; control: −0.52 ± 0.45 D/year, 0.32 ± 0.21 mm/year, n = 366.

**Table 2:** Effect on myopia progression after outdoor light exposure: Comparison between the intervention group and control group (both myopia and nonmyopia group).

	Intervention Types	Outdoor Light Exposure Time at School (h/week)	The Reduction in Myopia Incidence (%)	The Reduction in SER (%)	The Reduction in Axial Elongation (%)
Meta-Analysis			50%	32.9%	24.9%
Li et al., 2018_II [40]	Intervention Group II had 7 h/week of exposure and an extra 5 h/week after school.	12	69%	69.2%	62.5%
Wu et al., 2013 [37]	Total daily recess time was 80 min; total weekly recess time was 6.7 h. The control group did not have any special program during recess. Schools had 2 h of outdoor physical education per week.	8.7	64%	34.2%	
Li et al., 2018_I [40]	Intervention Group I had 7 h/week of exposure, including recess and physical education.	7	50%	23.1%	25%
Wu et al., 2018 [39]	If children went outside the classroom during every recess, they would accumulate 200 min of outdoor time per 5-day school week.	3.3	35%	25.5%	15.2%
He et al., 2015 [29]	An additional 40-min outdoor light exposure class was scheduled at the end of each school day. The study did not explain class recess time.	Unclear	24%	10.1%	4.7%
Jin et al., 2015 [38]	The interventions were two additional 20-min recesses programs for outdoor light exposure. The study did not explain class recess time.	Unclear	58%	63%	23.8%
Yi & Li, 2011 [36]	The children in the intervention group had near and middle vision exposure of >30 h/week and more outdoor light exposure than 14–15 h/week. The study did not explain class recess time.	Unclear		26.9%	

## Supplementary Materials

The following materials are available online at https://www.mdpi.com/1660-4601/16/14/2595/s1, Table S1: Assessment of methodological quality for cross-sectional studies; Table S2: Assessment of methodological quality for cohort studies; Figure S1: Risk of bias summary for intervention studies; Figure S2: Risk of bias chart for intervention studies.

## References

[1] Pan C.W., Ramamurthy D., Saw S.M. (2012). Worldwide prevalence and risk factors for myopia. Ophthalmic Physiol. Opt. J. Br. Coll. Ophthalmic Opt..

[2] Dolgin E. (2015). The myopia boom. Nature.

[3] Morgan I.G., Ohno-Matsui K., Saw S.M. (2012). Myopia. Lancet.

[4] Holden B.A., Fricke T.R., Wilson D.A., Jong M., Naidoo K.S., Sankaridurg P., Wong T.Y., Naduvilath T.J., Resnikoff S. (2016). Global prevalence of myopia and high myopia and temporal trends from 2000 through 2050. Ophthalmology.

[5] Marcus M.W., de Vries M.M., Junoy Montolio F.G., Jansonius N.M. (2011). Myopia as a risk factor for open-angle glaucoma: A systematic review and meta-analysis. Ophthalmology.

[6] Flitcroft D.I. (2012). The complex interactions of retinal, optical and environmental factors in myopia aetiology. Prog. Retin. Eye Res..

[7] Saw S.M., Gazzard G., Shih-Yen E.C., Chua W.H. (2005). Myopia and associated pathological complications. Ophthalmic Physiol. Opt. J. Br. Coll. Ophthalmic Opt..

[8] Dudovitz R.N., Izadpanah N., Chung P.J., Slusser W. (2016). Parent, teacher, and student perspectives on how corrective lenses improve child wellbeing and school function. Matern. Child Health J..

[9] Kumaran S.E., Balasubramaniam S.M., Kumar D.S., Ramani K.K. (2015). Refractive error and vision-related quality of life in South Indian children. Optom. Vis. Sci. Off. Publ. Am. Acad. Optom..

[10] Holden B., Sankaridurg P., Smith E., Aller T., Jong M., He M. (2014). Myopia, an underrated global challenge to vision: Where the current data takes us on myopia control. Eye.

[11] Lim M.C., Gazzard G., Sim E.L., Tong L., Saw S.M. (2009). Direct costs of myopia in Singapore. Eye.

[12] Jones L.A., Sinnott L.T., Mutti D.O., Mitchell G.L., Moeschberger M.L., Zadnik K. (2007). Parental history of myopia, sports and outdoor activities, and future myopia. Investig. Ophthalmol. Vis. Sci..

[13] Rose K.A., Morgan I.G., Ip J., Kifley A., Huynh S., Smith W., Mitchell P. (2008). Outdoor activity reduces the prevalence of myopia in children. Ophthalmology.

[14] Wu P.C., Tsai C.L., Hu C.H., Yang Y.H. (2010). Effects of outdoor activities on myopia among rural school children in Taiwan. Ophthalmic Epidemiol..

[15] Guggenheim J.A., Northstone K., McMahon G., Ness A.R., Deere K., Mattocks C., Pourcain B.S., Williams C. (2012). Time outdoors and physical activity as predictors of incident myopia in childhood: A prospective cohort study. Investig. Ophthalmol. Vis. Sci..

[16] Guo K., Yang D.Y., Wang Y., Yang X.R., Jing X.X., Guo Y.Y., Zhu D., You Q.S., Tao Y., Jonas J.B. (2015). Prevalence of myopia in schoolchildren in Ejina: The Gobi desert children eye study. Investig. Ophthalmol. Vis. Sci..

[17] Cohen Y., Belkin M., Yehezkel O., Solomon A.S., Polat U. (2011). Dependency between light intensity and refractive development under light-dark cycles. Exp. Eye Res..

[18] Ashby R.S., Schaeffel F. (2010). The effect of bright light on lens compensation in chicks. Investig. Ophthalmol. Vis. Sci..

[19] Norton T.T., Siegwart J.T. (2013). Light levels, refractive development, and myopia-a speculative review. Exp. Eye Res..

[20] Feldkaemper M., Schaeffel F. (2013). An updated view on the role of dopamine in myopia. Exp. Eye Res..

[21] Ashby R., Ohlendorf A., Schaeffel F. (2009). The effect of ambient illuminance on the development of deprivation myopia in chicks. Investig. Ophthalmol. Vis. Sci..

[22] McCarthy C.S., Megaw P., Devadas M., Morgan I.G. (2007). Dopaminergic agents affect the ability of brief periods of normal vision to prevent form-deprivation myopia. Exp. Eye Res..

[23] Sherwin J.C., Reacher M.H., Keogh R.H., Khawaja A.P., Mackey D.A., Foster P.J. (2012). The association between time spent outdoors and myopia in children and adolescents: A systematic review and meta-analysis. Ophthalmology.

[24] Xiong S., Sankaridurg P., Naduvilath T., Zang J., Zou H., Zhu J., Lv M., He X., Xu X. (2017). Time spent in outdoor activities in relation to myopia prevention and control: A meta-analysis and systematic review. Acta Ophthalmol..

[25] Higgins J., Green S. (2011). Cochrane Handbook for Systematic Reviews of Interventions.

[26] Ottawa Hospital Research Institute. http://www.ohri.ca/programs/clinical_epidemiology/oxford.asp.html.

[27] Herzog R., Álvarez-Pasquin M.J., Díaz C., Del Barrio J.L., Estrada J.M., Gil Á. (2013). Are healthcare workers’ intentions to vaccinate related to their knowledge, beliefs and attitudes? A systematic review. BMC Public Health.

[28] Chinn S. (2000). A simple method for converting an odds ratio to effect size for use in meta-analysis. Stat. Med..

[29] He M., Xiang F., Zeng Y., Mai J., Chen Q., Zhang J., Smith W., Rose K., Morgan I.G. (2015). Effect of time spent outdoors at school on the development of myopia among children in China: A randomized clinical trial. Jama.

[30] Donovan L., Sankaridurg P., Ho A., Naduvilath T., Smith E.L., Holden B.A. (2012). Myopia progression rates in urban children wearing single-vision spectacles. Optom. Vis. Sci. Off. Publ. Am. Acad. Optom..

[31] Ip J.M., Saw S.M., Rose K.A., Morgan I.G., Kifley A., Wang J.J., Mitchell P. (2008). Role of near work in myopia: Findings in a sample of Australian school children. Investig. Ophthalmol. Vis. Sci..

[32] Guo Y., Liu L.J., Xu L., Lv Y.Y., Tang P., Feng Y., Meng M., Jonas J.B. (2013). Outdoor activity and myopia among primary students in rural and urban regions of Beijing. Ophthalmology.

[33] Zhou R., Zhang W.F., Yang Y., Li Y.T., Zhang J., Wang W.P. (2014). Analysis of myopia prevalence and influencing factors among primary school students in the urban area of Lanzhou city. Int. Eye Sci..

[34] Zhou Z., Morgan I.G., Chen Q., Jin L., He M., Congdon N. (2015). Disordered sleep and myopia risk among Chinese children. PloS ONE.

[35] French A.N., Morgan I.G., Mitchell P., Rose K.A. (2013). Risk factors for incident myopia in Australian schoolchildren: The Sydney adolescent vascular and eye study. Ophthalmology.

[36] Yi J.H., Li R.R. (2011). Influence of near-work and outdoor activities on myopia progression in school children. Chin. J. Contemp. Pediatrics.

[37] Wu P.C., Tsai C.L., Wu H.L., Yang Y.H., Kuo H.K. (2013). Outdoor activity during class recess reduces myopia onset and progression in school children. Ophthalmology.

[38] Jin J.X., Hua W.J., Jiang X., Wu X.Y., Yang J.W., Gao G.P., Fang Y., Pei C.L., Wang S., Zhang J.Z. (2015). Effect of outdoor activity on myopia onset and progression in school-aged children in northeast China: The Sujiatun eye care study. BMC Ophthalmol..

[39] Wu P.C., Chen C.T., Lin K.K., Sun C.C., Kuo C.N., Huang H.M., Poon Y.C., Yang M.L., Chen C.Y., Huang J.C. (2018). Myopia prevention and outdoor light intensity in a school-based cluster randomized trial. Ophthalmology.

[40] Li J., Liu F., Zhou X.W., Li C.G., Huang H.H. (2018). Effect of outdoor exposure on myopia prevention among school-aged children. Chin. J. Sch. Health.

[41] Dirani M., Tong L., Gazzard G., Zhang X., Chia A., Young T.L., Rose K.A., Mitchell P., Saw S.M. (2009). Outdoor activity and myopia in Singapore teenage children. Br. J. Ophthalmol..

[42] Liao C.C., Chen L.J., Yu J.H., Lin J.C. (2014). Refractive error change and its association with ocular and general parameters in junior high school students in Taiwan. Jpn. J. Ophthalmol..

[43] Gwiazda J., Hyman L., Hussein M., Everett D., Norton T.T., Kurtz D., Leske M.C., Manny R., Marsh-Tootle W., Scheiman M. (2003). A randomized clinical trial of progressive addition lenses versus single vision lenses on the progression of myopia in children. Investig. Ophthalmol. Vis. Sci..

