# Supplementary material for: Dose–Response Relationship of Outdoor Exposure and Myopia Indicators: A Systematic Review and Meta-Analysis of Various Research Methods
Source: Int J Environ Res Public Health. 2019 Jul 21;16(14):2595. doi: 10.3390/ijerph16142595 (PMC6678505; doi:10.3390/ijerph16142595)
Supplement: Supplementary file 1 [file ijerph-16-02595-s001.pdf]

SUPPLEMENTARY MATERIAL

Table.S1. Assessment of methodological quality for cross-sectional studies.

Author, year	Selection	Comparability	Exposure/Outcome	Total scores
Zhou et al., 2014 [33]	****	*	***	8/10
Zhou et al., 2015 [34]	****	*	***	9/10
Guo et al., 2013 [16]	****	*	***	8/10
Ip et al., 2008 [31]	****	*	***	8/10

Table.S2. Assessment of methodological quality for cohort studies.

Author, year	Selection	Comparability	Exposure/Outcome	Total scores
French et al. 2013 [35]	***	*	***	7/9
Guggenheim et al., 2012 [15]	***	*	***	7/9
Jones et al., 2007 [12]	***	*	**	6/9

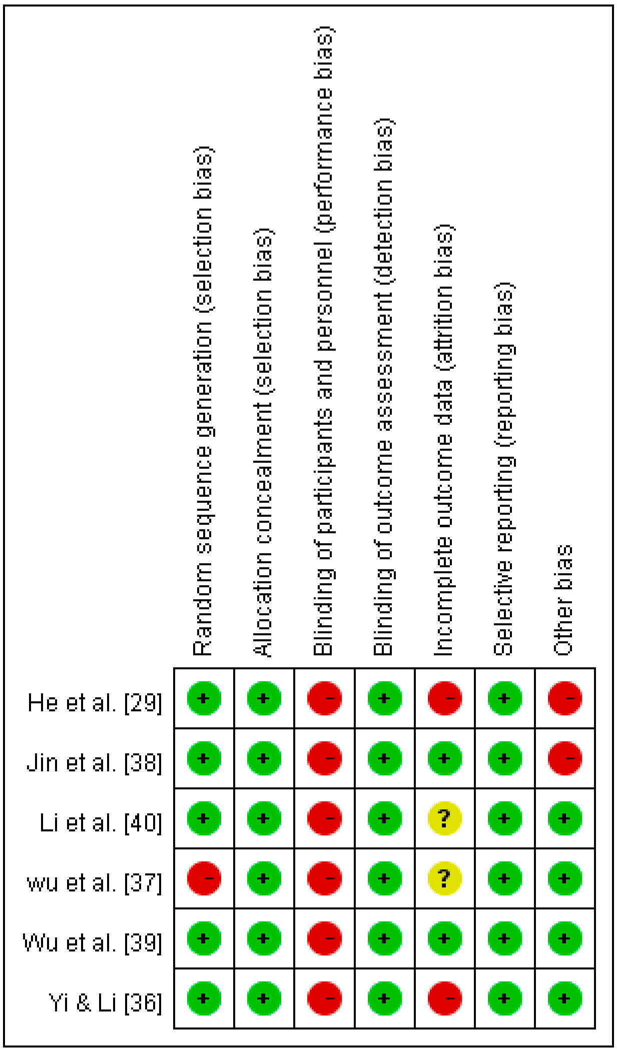

Figure.S1. Risk of bias summary for intervention studies.

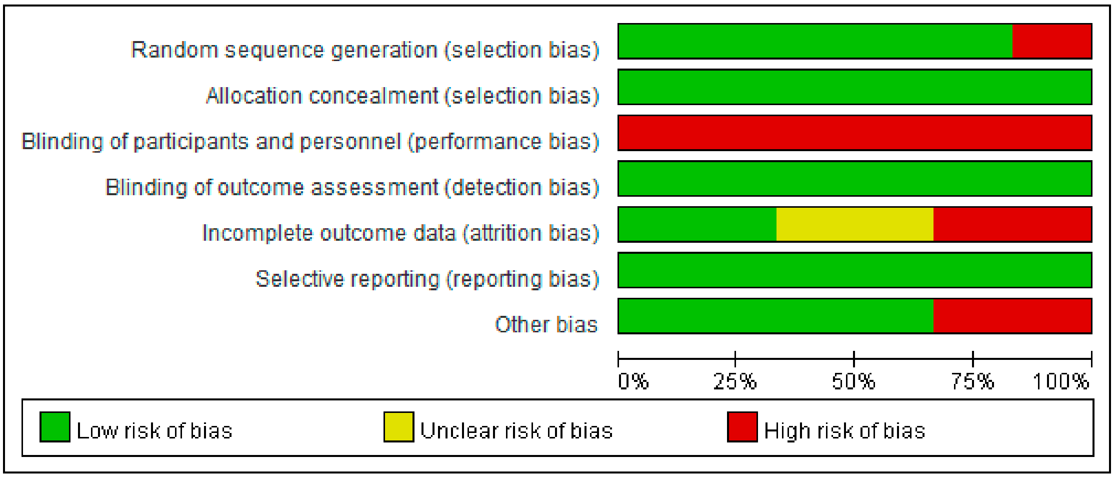

Figure.S2. Risk of bias chart for intervention studies.
